# Characterizing the syphilis epidemic among men who have sex with men in Lima, Peru to identify new treatment and control strategies

**DOI:** 10.1186/1471-2334-13-426

**Published:** 2013-09-10

**Authors:** Robert G Deiss, Segundo R Leon, Kelika A Konda, Brandon Brown, Eddy R Segura, Jerome T Galea, Carlos F Caceres, Jeffrey D Klausner

**Affiliations:** 1 Division of Infectious Diseases, Harbor-UCLA Medical Center, 1000 W. Carson Street, Box 466, Torrance, CA 90502, USA; 2 Program in Global Health, Division of Infectious Diseases, David Geffen School of Medicine, University of California, 10833 Le Conte Ave. CHS 12-105, Los Angeles, CA 90095-1688, USA; 3 Unit of Health, Sexuality and Human Development, Cayetano Heredia University School of Public Health, Lima, Peru. Av. Armendariz 445 Lima 18, Peru; 4 Program in Public Health, Department of Population Health & Disease Prevention, University of California, Irvine, 653 E. Peltason Road Suite 2010, Irvine, CA 92697-3957, USA; 5 Department of Global Health, University of Washington, Harborview Medical Center, 325 9th Avenue Box 359931, Seattle, WA 98104, USA; 6 Centre for Sexual Health and HIV Research, Faculty of Population Health, University College London, Gower St, London, Greater London WC1E 6BT, UK

**Keywords:** Syphilis, Peru, Men who have sex with men (MSM), HIV, T. pallidum, Molecular epidemiology, Cytokine, Macrolide resistance

## Abstract

**Background:**

Syphilis is an important sexually transmitted infection (STI) with serious public health consequences. Among men who have sex with men (MSM) in Lima, the prevalence and incidence are extraordinarily high. Current syndromic approaches, however, fail to identify asymptomatic cases, and in settings where large proportions of individuals test positive again after treatment, it is frequently difficult to distinguish treatment failure from re-infection. Thus, new approaches are needed to improve treatment strategies and public health control efforts.

**Methods/Design:**

Study participants will undergo baseline testing for syphilis infection along with a behavioral survey covering demographics, sexual behavior, drug and alcohol abuse and health-care seeking behavior. The cohort will be followed for 18 months at three-month intervals. Blood and earlobe scrapings will also be collected for *T. pallidum* DNA testing, to create molecular markers for subtyping. We will also perform cytokine testing on collected samples in order to create host immunologic profiles associated with recurrence, re-infection, treatment failure and success.

**Discussion:**

Advances in social epidemiology, molecular typing and characterization of host immune responses will offer promise in developing new understandings of syphilis management. We will share our findings with the Peruvian Ministry of Health and other public health organizations, to identify new approaches of case detection and successful treatment.

## Background

Syphilis remains an important sexually transmitted infection (STI) with grave medical and public health consequences if left untreated. It is caused by the bacterium *Treponema pallidum (T. pallidum)* and is the leading cause of preventable infant mortality, surpassing HIV infection globally [1]. The risk of acquiring and transmitting HIV infection is increased in the presence of syphilitic ulcers [2,3]. Between 10 and 12 million new infections of syphilis occur worldwide annually, including an estimated 2–3 million cases in Latin America, [4] mostly among high-risk populations including sex workers, men who have sex with men (MSM) and male-to-female transgender women (TW) [5].

Despite existing prevention and control programs, syphilis infection remains an important public health problem in Lima, Peru, concentrated largely among MSM and TW [6-8]. A study of 1,056 high-risk MSM/TW found a lifetime prevalence of 21.6% and an incidence rate of 8.4 cases/100 person-years (95% CI: 6.7-7.1) [7]. A related study found that among HIV-antibody positive MSM/TW, the prevalence of active, untreated disease was 21% and a lifetime history of syphilis infection was 35.5% [9]. High rates of syphilis infection among MSM/TW have implications for the broader population in Peru, as previous studies have found higher prevalence of syphilis among men reporting same-sex behavior [10] and among women whose male partners had sex with men [11,12].

In Peru and other Latin American countries, the current syndromic approach to syphilis management, which relies on diagnosis and treatment based on recognition of ulcerative lesions, has proven inadequate in controlling the syphilis epidemic, and thus, new approaches are warranted. Syphilis control efforts are frequently complicated by treatment failure and high rates of repeat infection—up to 43% in one study in Peru, [13] and failure to detect asymptomatic infections [14]. Often, it is not clear whether a new diagnosis represents past treatment failure, re-infection or identification of a previously asymptomatic infection, and thus, improved case-finding and diagnostic strategies are urgently needed.

**Figure 1:**
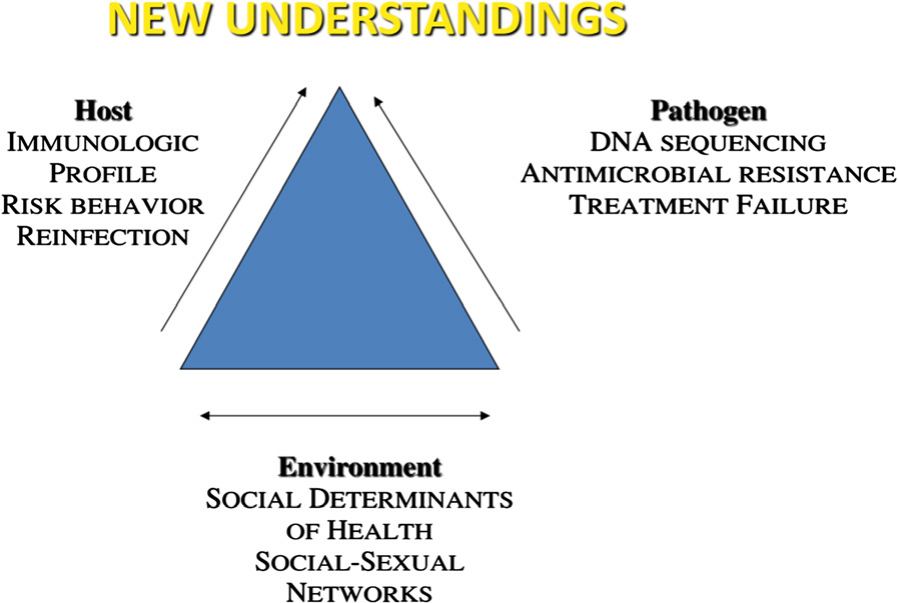
**Our study invokes the classic disease triangle through a) measurement of cytokines and immune responses (HOST), DNA sequencing of T. pallidum (PATHOGEN) and our survey of social determinants and sexual risk behaviors to elucidate social-sexual networks (ENVIRONMENT).** Interplay between these domains will inform new strategies for syphilis control efforts.

In order to characterize the determinants of the current syphilis epidemic among MSM/TW in Peru, and to better inform syphilis treatment and control strategies worldwide, it is critical to study various aspects of the pathogen, host, and environment among syphilis cases. Here we describe an observational study focused on MSM/TW who were diagnosed with, or are at high risk of syphilis, that will take place over a period of 5 years (2013–2017) in Lima, Peru. We will first characterize the prevalence and incidence of syphilis among MSM/TW. Next, through ascertainment of treatment status, host immune response, and pathogen genotype analysis, we will classify cases as reinfection, persistent infection/treatment failure, or recurrence based on incomplete treatment. Data will be collected on diagnosis/treatment history, sexual behavior, and in-depth immunological and molecular biologic aspects of the pathogen. To improve the population-specific understanding of syphilis, molecular typing will provide broad information on the *T. pallidum* bacteria encountered to distinguish between re-infection with a new strain, antimicrobial resistance or persistence of original infection. Through behavioral, diagnostic, molecular and immunological research, our study offers potential to develop new approaches that may inform strategies for improving current syphilis control strategies (Figure 1).

## Methods/Design

### Overview

Our group, led by a collaboration between experienced senior investigators from Universidad Peruana Cayetano Heredia (UPCH) and University of California, Los Angeles (UCLA), Epicentro and Barton Health Center will study a cohort of high-risk MSM/TW. The cohort is expected to have a baseline prevalence of 10% for untreated syphilis, an annual incident rate of 5–10%, and treatment failure rates or re-infection of 25–50% [7,14]. Studying syphilis in this population provides several opportunities: 1) ready identification of primary and secondary stages of syphilis, making clinical specimens of *T. pallidum* available for molecular characterization; 2) description of outcomes including treatment success, failure or re-infection in a short observation period; and 3) development of a cohort to allow frequent monitoring of behavioral, clinical and immunological parameters to define predictors of infection, treatment response, and re-infection. The study protocol has been approved by the institutional review boards at UPCH, Barton Health Center and UCLA.

Our cohort will consist of 400 MSM/TW meeting high risk criteria for syphilis, who will be assessed at baseline and then at 3, 6, 9, 12, 15 and 18 months. Study visits will entail behavioral surveys and testing for syphilis and other STIs. We will also obtain samples for cytokine measurements and to test for T. pallidum DNA, as detailed below, in order to understand molecular and immune profiles associated with markers of treatment success and failure.

### Participant recruitment and retention

Between 40–60 participants will be recruited each month from two clinical sites in Lima, including a public reference STI clinic (Barton Health Center) specialized in at-risk populations including MSM/TW, and a Gay Men’s community center (Epicentro) located in a metropolitan area of Lima. Both locations are well-recognized and reachable by the target population, particularly lower-middle income individuals. We will screen up to 1,000 patients in order to recruit a high-risk cohort of 400 people. We project that Barton Health Center will account for 70% of the recruited patients, with 30% recruited at Epicentro.

Men who are 18 years or older will be invited to join the two-year study if they meet at least three of the following high-risk criteria: i) having had a positive syphilis test in the past 2 years, ii) having HIV infection, iii) having any syndromic ulcer-related STI (i.e. HSV-2, syphilis or genital ulcerative disease) at the time of screening; iv) having been diagnosed with an STI in the past 6 months); v) having more than 5 years of sexual activity; vi) having more than 5 sex partners in the past 3 months, or vii) having more than 5 occurrences of unprotected anal sex in the past 6 months.

To facilitate participant retention and follow-up care, we will utilize cell phone text messages (SMS), phone calls, e-mail and Internet chat to provide regular follow-up reminders. Several studies have shown that text reminders, provided on a weekly basis, increase cohort retention and follow-up [15,16]. The use of mobile phones and internet has increased dramatically in Peru in the last several years and is a preferred way of communication, allowing for novel methods for STI management, case-finding, treatment follow-up and cohort retention [17].

Study visits will be held within established clinical settings, which will provide safe and private spaces for conducting surveys, obtaining samples and providing clinical care. At the time of enrollment, participants will meet individually with a staff member to review all study procedures, including the benefits and risks of participation, the option to discontinue participation at any time without penalty, and means of contacting the investigator and the local study institutional review board if questions about participation in the study arise. Signed informed consent will be obtained from all participants prior to any study activities. Study forms will include de-identified numbers, and data from all encounters will be maintained in locked areas. Study information sheets with personally identifying contact information will be stored in separate locked areas, with linkage information only available to the study investigators.

### Data collection and laboratory testing

Demographic characteristics, sexual and risk behavior, health/health care seeking behavior, history of current/past syphilis infection (including treatment), alcohol use and substance abuse history will be assessed at baseline and three month intervals. Follow-up risk behavior assessments will elicit self-reported behavior for the last three months only. Participants will receive pre-test counseling along with a rapid HIV test (with EIA and Western Blot confirmation). Participants diagnosed with HIV infection will be referred to the Peruvian National HIV Treatment Program. Syphilis testing will be performed via sampling of whole blood, lesion swabs (if present) and ear lobe scraping (when rapid syphilis testing is positive and/or lesions are present) [18,19].

We will utilize a highly accurate point-of-care specific test to identify antibody-positive patients [20]. Syphilis-specific *T. pallidum* antibody tests are more sensitive and specific for syphilis infection than conventional non-treponemal tests used in routine clinical practice, particularly in early infection [21]. Rapid Plasma Reagin (RPR) tests will be used to establish specific titers followed by confirmation through *T. pallidum*-Particle Agglutination test (TPPA) using a cutoff value of ≥ 1:80. Treatment for syphilis will be offered on-site for detected cases, in line with Peruvian National STI Treatment Guidelines, [22] (see Table 1). Individuals with neurologic symptoms will be referred for additional diagnostics and care.

**Table 1:** Peruvian syphilis treatment guidelines (non-pregnant)

	**Treatment**	**Alternative**
Primary	Penicillin G 2.4 million Units IM, single dose	Doxycyline 100 mg po bid × 2 weeks^1^
Secondary	Penicillin G 2.4 million Units IM, single dose	Doxycyline 100 mg po bid × 2 weeks^1^
Early latent	Penicillin G 2.4 million Units IM, single dose	Doxycyline 100 mg po bid × 2 weeks^1^
Late latent	Penicillin G 2.4 million Units IM, 3 weekly doses	Doxycyline 100 mg po bid × 4 weeks^1^
Tertiary	Penicillin G 2.4 million Units IM, 3 weekly doses	Doxycyline 100 mg po bid × 4 weeks^1^
Neurosyphilis	Penicillin G 4 million units IV q4h × 10–14 days	Penicillin Procaine 2.4 million units IM daily × 10–14 days with Probenecid 500 mg po four times daily × 10–14 days

^1^Tetracycline 500 mg po qid may be substituted for Doxycycline.

We will collect three types of samples: lesion exudates (if lesions are found), blood samples and ear lobe scrapings for *T. pallidum* DNA detection, previously unmeasured together in any cohort study. Individuals who test positive for *Treponema pallidum* DNA by these methods will be treated in the same manner as the RPR/TPPA algorithm described above. In quarterly follow-up visits, individuals who were not reactive to rapid syphilis tests at recruitment will be assessed for new syphilis infections with repeat serologic tests; those who were diagnosed with syphilis at recruitment or subsequent visits will be assessed for cure, or re-infection/persistence of seroreactivity.

### Sample size and power calculations

Based on our research experience we anticipate screening about 600 potential participants in order to reach 400 eligible under the current high-risk eligibility requirements. A sample size of 400 will produce a two-sided 95% confidence interval with a width equal to 8 units when the actual baseline population syphilis prevalence equals 20% [14]. With a potential attrition rate as extreme as 18% over 18 months from our previous research experience, [16] the least expected final sample size for the cohort analysis will be 328. Extrapolating a previously reported incidence rate of 8.4 per 100 person-years in similar high-risk Peruvian MSM populations [7] to our final sample of 328, and assuming homogenous occurrence of incident syphilis cases during the proposed 18 months of follow-up, we expect 41 total incident cases and a cumulative incidence of 12.5%, with a 95% confidence interval width equal to 7%. All sample size estimations were computed using NCSS/PASS 2011 software (Kaysville, UT, 2011).

### Statistical analyses

Frequency distributions will be tabulated for all variables, performing range checks and cross-validations, and individually evaluating all data points with measurements three standard deviations above their mean. Missing or erroneous data will be coded as such and frequencies of unusable data will be calculated for every variable to determine if the frequency was unusually high and affected the validity of the data. All outcomes will be calculated at the individual level: 1) number of prevalent syphilis cases in the initially screened population, 2) number of incident syphilis cases during the study period, and 3) number of persistent syphilis infections based on RPR titers during the study period. Observation time will be adjusted for subjects who do not complete the study follow-up.

New cases and individuals who received treatment at baseline but who have a 4-fold titer increase at follow-up visits will be considered incident cases. The rate will be expressed as incidence density, calculated with the total number of incident STIs and total observation time. In addition to the incidence and prevalence of syphilis infection, we will also calculate the number of persistent syphilis infections as defined by participants with prevalent or incident cases of syphilis whose RPR titers do not decrease adequately over time.

Bivariate and multivariate analyses will be conducted to determine socio-behavioral and biological variables associated with all three outcomes of interest listed above. For the prevalent syphilis outcome, prevalence ratios will be calculated to avoid overestimation of measures of association which would result from logistic regression [23]. For the additional outcomes (incident and persistent syphilis), incidence rate ratios with confidence intervals will be calculated, and Cox regression will be used for the multivariate models. A sequential approach using nested models will be used to determine the statistical significance of the variables, using the likelihood ratio test to assess the strength of the evidence against the null hypothesis of no-effect for each variable.

### Molecular analysis

Detection of *T. pallidum* DNA has been facilitated by improved methods of collection and amplification [18,19,24-27]. To optimize the detection of *T. pallidum* DNA in our study, we will swab any suspicious clinical lesions, collect whole blood samples by venipuncture and from ear lobe scrapings, and conduct routine anoscopy and dark-field examination of mucosal lesions among participants at every three-month visit. While clinical lesions like anogenital ulcers provide the best source of *T. pallidum* DNA, newer work has shown that detection of *T. pallidum* DNA in whole blood from ear lobe scrapings is more sensitive than venous whole blood when no lesions are present[18,19]. In prior studies, *T. pallidum* detection ranged from 70–90% in lesions of primary and secondary syphilis and 30–50% in whole blood collected by venipuncture. In latent syphilis infection, Castro et al. were able to demonstrate *T. pallidum* DNA from 56–76% of whole blood specimens collected by ear lobe scraping [19]. From these samples, we will use conventional PCR to amplify the gene region of *polA*, the most common target of PCR amplification for *T. pallidum* DNA [28]. As a control for specimen adequacy, DNA extraction and the detection of PCR inhibitors, we will also amplify human β–globin gene in all clinical specimens.

In addition to improvements in DNA extraction, the molecular characterization of *T. pallidum* has evolved in recent years to newer, more discriminatory methods which use multiple genes and targets. Molecular subtyping has also been performed on *T. pallidum* specimens, allowing researchers to link sub-types to specific transmission networks or clinical outcomes, [29] including neurosyphilis. As the treatment for neurosyphilis differs from the treatment of genital ulcer disease or clinical syphilis, the characterization of *T. pallidum* subtype could influence syphilis diagnosis and management in Peru.

The development of molecular markers of cure is also an evolving and promising area of research. In Castro’s study of *T. pallidum* DNA collected via sera, whole blood, plasma or earlobe scrapings, no patients with treated syphilis (n = 18) had detectable *T. pallidum* DNA, thus providing one of the first demonstrations of a microbiological marker for treatment success of latent syphilis in humans [19]. Blood specimens collected from ear lobe scrapings have also been used to demonstrate microbiologic clearance among patients treated for latent syphilis [14]. We will therefore combine conventional serological markers with clinical response to therapy and microbiological clearance as determined by the presence or absence of *T. Pallidum* DNA. By using that microbiological marker of treatment success, we hope to identify serum cytokine profiles associated with microbiologic cure. As serum markers are more readily collected and measured than ear lobe blood specimens, this avenue of research could provide a major advance in syphilis therapeutic monitoring and cure.

### Antimicrobial resistance

Our PCR–based assay will also be used to screen for the 23S rRNA gene mutation to macrolides. This point mutation is important in altering susceptibility to treatment with azithromycin, [24] resulting in high incidence of clinical treatment failure. There have been no studies of azithromycin resistance to *T. pallidum* in Peru, and while not widely used, epidemiologic surveillance is of substantial public health importance to inform and update current national treatment guidelines, particularly for individuals who are penicillin-allergic or in whom tetracyclines are contraindicated (e.g. children and pregnant women). Knowledge from antimicrobial resistance classification will further help determine whether treatment failures are due to infection with an antimicrobial resistant pathogen, non-adherence, or host immune system failure.

### Immunologic basis of syphilis infection

Measurement of cytokine activity in clinical blood specimens has been revolutionized with the advent of Luminex technology. Knudsen et al. recently showed that early stage infection was associated with an increase in IL-10 whereas IL-10 and TNF-a both decreased after treatment of syphilis [30]. These clinical data build on prior work showing how Th1 and Th2 cytokine patterns corresponded to disease progression, [31] as IL-10 has further been postulated to play an important role in dampening the host’s response to infection [32]. Similarly, in an animal model of another spirochetal infection, *Borrelia spp,* IL-10 deficiency was associated with rapid innate immune clearance of infection [33]. Thus, persistent expression of IL-10 in humans might be associated with treatment failure and/or re-infection.

In order to identify potential markers for cure, we will determine serum cytokine profiles of patients before, during and after syphilis infection and associate these profiles with various categories of patient outcomes, including treatment success/failure and re-infection. Additionally, we will compare these profiles with profiles from uninfected patients as a control group, to identify predictors of infection and response to therapy. We hypothesize that in early infection, we will observe a dominant Th2 response (increased IL-10) that evolves to an effective Th1 response (IL-2, IFN-gamma, TNF-alpha) associated with the clearance of infection.

In addition, cytokines related to Th1 immunity will be assessed in conjunction with serologic RPR titers, presence of lesions if any, re-infection, serological persistence and *T. pallidum* sub-species. To inform the development of new diagnostics, we will build a predictive cytokine model associated with new and persistent infection with syphilis. Given the number of cytokines of interest, we will apply Sidak’s correction for multiple comparisons to adjust p-values defining significance. We will then perform bivariate and multivariate analyses, using likelihood ratio testing to determine inclusion variables. In addition, receiver-operating characteristic (ROC) and calibration curves will be used to determine the accuracy of the model. Depending on the sample size, the model may be built on half of the data and reproduced on the remaining half to explore transportability [34].

## Discussion

We anticipate that the benefits of study participation will outweigh risks, including loss of confidentiality and privacy of clinical information. A number of mechanisms will be enforced to minimize these risks: In turn, participants will benefit from treatment of newly-diagnosed STIs, along with increased awareness and education pertinent to STI prevention. New understandings obtained from this study will also provide indirect benefit to through impact and potential improvement of treatment norms and guidelines.

It is critical that all participants with positive syphilis tests at any follow-up point be treated. At the time of enrollment, participants will be asked to provide contact information such as a name or nickname, address and phone number, email address, and telephone number, as well as the names and contact information for two relatives or friends that will know their whereabouts, although participants will not be excluded from the study if they do not wish to provide additional contacts. For positive test results obtained after point-of-care testing and RPR titration (e.g. Western Blot and/or CT/NG diagnosis), the study team will contact participants by telephone or text message and set up an appointment for results notification and no-cost treatmentor referral to the Peruvian National HIV Treatment Program for HIV treatment.

General findings and recommendations for changes in STI management guidelines will be presented to, and discussed with, two different constituencies: (a) a technical group (other researchers, government officials, infectious disease and sexual health specialists); and (b) a community group (community advisory board and other community representatives). A final version of recommendations will be provided to key stakeholders, community members, and the Ministry of Health of Peru. We will make great efforts to identify feasible changes and produce concrete recommendations that can be proposed for revision of the present syphilis management guidelines, particularly in Peru. Our study will also have relevance to other Latin American and lower-middle income countries, particularly those with concentrated epidemics among high-risk populations, such as MSM.

The information we intend to gather during the proposed study has the potential to provide a more comprehensive and sound understanding of the syphilis epidemic, including prevention, diagnosis, and treatment. Knowledge from the behavioral, diagnostic and molecular components of our study will offer potential to develop new approaches and inform efforts in syphilis control. Identification of pathogen subtypes will provide researchers the ability to distinguish intrinsic resistance and actual re-infection. Insights from the immunologic study of cytokines may provide new avenues in identifying potential serologic markers for cure. Thus, our study will help expand the current clinical and public health response to syphilis, which has proven insufficient in combating the epidemic, with the goal of ultimately reducing the incidence and prevalence of syphilis infection in Peru and worldwide.

## Competing interests

The authors declare that they have no competing interests.

## Authors’ contributions

RD drafted the manuscript and contributed to survey instrument design. SL contributed to study design and project implementation and assisted in manuscript revision. KK contributed to study design, drafted the survey instrument. BB contributed to study design and implementation. ES performed statistical analyses for the study. JG participated in early study design. CC and JK are principal investigators for the study and critically revised the manuscript. All authors read and approved the final manuscript.

## Pre-publication history

The pre-publication history for this paper can be accessed here:

http://www.biomedcentral.com/1471-2334/13/426/prepub
